# Clinical Outcomes of Thirteen Patients with Acute Chagas Disease Acquired through Oral Transmission from Two Urban Outbreaks in Northeastern Brazil

**DOI:** 10.1371/journal.pntd.0000711

**Published:** 2010-06-15

**Authors:** Claudilson J. C. Bastos, Roque Aras, Gildo Mota, Francisco Reis, Juarez Pereira Dias, Robson Silva de Jesus, Miralba Silva Freire, Eline G. de Araújo, Juliana Prazeres, Maria Fernanda Rios Grassi

**Affiliations:** 1 Medicine and Human Health PhD Program, Bahian School of Medicine and Public Health, Bahia, Brazil; 2 Edgard Santos University Hospital, Federal University of Bahia, Bahia, Brazil; 3 Epidemiological Surveillance Department of the Bahia Health Secretary, Bahia, Brazil; 4 Couto Maia Hospital, Bahia, Brazil; 5 Oswaldo Cruz Foundation-FIOCRUZ, Bahia, Brazil; New York University School of Medicine, United States of America

## Abstract

**Background:**

Outbreaks of orally transmitted *Trypanosoma cruzi* continue to be reported in Brazil and are associated with a high mortality rate, mainly due to myocarditis.

**Methods:**

This study is a detailed report on the disease progression of acute Chagas disease in 13 patients who were infected during two micro-outbreaks in two northeastern Brazilian towns. Clinical outcomes as well as EKG and ECHO results are described, both before and after benznidazole treatment.

**Results:**

Fever and dyspnea were the most frequent symptoms observed. Other clinical findings included myalgia, periorbital edema, headache and systolic murmur. Two patients died of cardiac failure before receiving benznidazole treatment. EKG and ECHO findings frequently showed a disturbance in ventricular repolarization and pericardial effusion. Ventricular dysfunction (ejection fraction <55%) was present in 27.3% of patients. After treatment, EKG readings normalized in 91.7% of patients. Ventricular repolarization abnormalities persisted in 50% of the patients, while sinus bradycardia was observed in 18%. The systolic ejection fraction normalized in two out of three patients with initially depressed ventricular function, while pericardial effusion disappeared.

**Conclusions:**

Myocarditis is frequently found and potentially severe in patients with acute Chagas disease. Benznidazole treatment may improve clinical symptoms, as well as EKG and ECHO findings.

## Author Summary

Chagas disease is caused by a parasitic protozoan transmitted to humans by the contaminated feces of blood-feeding assassin bugs from the *Triatominae* subfamily. It may also be transmitted from mother to baby during pregnancy, by breastfeeding, blood transfusion or organ transplant. In rare cases, the disease can also be caused by accidental ingestion of contaminated food (sugar cane or *açaí* juice, drinking water, etc.). Acute Chagas disease often presents itself as a mononucleosis-like syndrome, with symptoms including fever, lymph node enlargement and muscle pain. The mortality rate of acute Chagas disease is high, mainly due to heart failure as a consequence of cardiac fiber lesions. There are few studies describing clinical outcomes and the disease progression of patients who receive therapeutic treatment, especially with regard to cardiac exam findings. In this report, the authors describe clinical findings from two micro-outbreaks occurring in impoverished towns in northeastern Brazil. Prior to receiving treatment, patient mortality rate was 28.6% in one of the outbreaks, and one pregnant woman experienced a spontaneous abortion due to the disease in the other outbreak. Most patients complained of fever, dyspnea, myalgia and periorbital edema. After receiving a two-month course of treatment, clinical symptoms improved and the number of abnormalities in cardiac exams decreased.

## Introduction

American trypanosomiasis, or Chagas disease, is a zoonotic protozoan disease caused by the haemoflagellate *Trypanosoma cruzi*. The disease is endemic throughout Central and South America where about 17 million people are estimated to be infected and 100 million are at risk of infection [1]. In Brazil, the overall prevalence of Chagas disease is 4.2% and in the northeast region the infection rate can reach more than 5.0% [1]–[3]. In endemic areas, the primary infection usually occurs in children aged 15 years and under. More than 99% of acute Chagas disease cases are asymptomatic or appear as a nonspecific febrile disease. However, in untreated patients with severe symptoms of acute Chagas disease, the mortality rate rises to about 5–10% [1]. Moreover, 30% of infected individuals can develop chronic symptoms of Chagas disease over a lifetime [4].

Vectorial transmission of Chagas disease has decreased over the last decade in Brazil. Other forms of transmission, such as blood transfusion, congenital, organ transplants, and laboratory accidents are reported sporadically [1], [4]. The oral-accidental transmission of *Trypanosoma cruzi* is becoming increasingly common.

Since 1965, several outbreaks, possibly caused by oral-accidental routes, mainly due to ingestion of food, fresh water, “açaí” (*Euterpe oleracea*) or sugar cane juice, have occurred in many Brazilian states, including Rio Grande do Sul, Amazonas, Amapá, Santa Catarina, and Bahia [5]–[14]. In other Latin American countries, oral transmission has also been reported [15]–[16]. Oral infection with *T. cruzi* is associated with a high mortality rate, usually in the first two weeks after infection [8]–[11]. Mortality is mainly due to acute congestive heart failure, myocarditis and meningoencephalitis [4], [11]. Hemorrhagic manifestations and severe gastritis have also been reported [6].

Myocarditis is present in 80% of patients presenting severe symptoms of acute Chagas disease [4], [16]–[17]. Electrocardiography (EKG) and echocardiography (ECHO) show alterations such as atrial fibrillation and pericardial effusion, which are associated with a poor prognosis [11], [17]–[19]. In this study, we describe the clinical outcomes of 13 patients, before and after benznidazole treatment, during two micro outbreaks of acute Chagas disease in two towns located in the State of Bahia in northeastern Brazil.

## Materials and Methods

### Study Area and Patients

The patients involved in this study came from two neighboring towns: Macaúbas (46,554 inhabitants) and Ibipitanga (13,109 inhabitants), both located in the south central region of the state of Bahia (approximately 700 km from the capital) in the Brazilian Northeast. In both towns, the annual per capita income is less than U$1,000 and the UN human development index is 0.62521. In May 2006, there was an outbreak of acute Chagas disease involving seven individuals from Macaúbas (cases 1–7) [8]. All individuals were members of the same family. Acute Chagas disease was suspected by a local physician and diagnosis was laboratory-confirmed in five cases (cases 1–5). Two patients (cases 6, 7) died as a consequence of heart failure before Chagas disease was confirmed. Oral contamination with *T. cruzi* probably occurred via ingestion of improperly stored water, possibly contaminated by feces of infested *T. sordida* [8].

The Ibipitanga outbreak occurred a few months after that of Macaúbas and involved six cases occurring among a family of 11. On August 9, 2006, the six cases: father (case 9), three sons (cases 10, 12, 13), one daughter (case 11) and his daughter-in-law (case 8) were working on a sugarcane plantation and drank a freshly-made sugarcane juice between 8:00 and 9:00 am, which they prepared in an abandoned sugarcane mill located next to the plantation. The cases developed symptoms between 11 and 21 days (between August 20 and 30, 2006) after the day they drank the sugar cane juice. A diagnosis of acute Chagas disease was suspected by a local physician 49 days after the ingestion of sugar cane juice (September 27, 2006). On October 8, 2006, the Epidemiological Surveillance Department of the Bahia Health Secretariat investigated the outbreak. Twelve specimens of *Triatoma sordida* were captured at the sugarcane mill, one of which was infested with *T. cruzi*. The diagnosis of Chagas disease was laboratory-confirmed for all six cases based on positive serological test results from samples collected on October 8, 2006, almost 60 days after exposure (Table 1).

**Table 1:** Serological test results from 13 patients with acute Chagas disease in two urban outbreaks Bahia, Brazil, 2006.

ID case	Parasitological test	IFAT (IgM)	ELISA (IgM)	ELISA recombinant antigens (IgM)
1	Negative	Positive	n/a	n/a
2	Positive	Positive	n/a	n/a
3	Positive	Positive	n/a	n/a
4	Positive	Positive	n/a	n/a
5	Negative	Positive	n/a	n/a
6	n/a	n/a	n/a	n/a
7	n/a	n/a	n/a	n/a
8	Negative	Positive	Positive	Positive
9	Negative	Positive	Positive	Positive
10	Negative	Positive	Positive	Positive
11	Negative	Positive	Positive	Positive
12	Negative	Positive	Positive	Positive
13	Negative	Positive	Positive	Positive

n/a: not available.

cases 1–5: samples collected on May 5, 10 and 15, 2006 (almost 30 days after exposure); cases 6,7: no samples collected (patients died before Chagas disease was confirmed) [8].

cases 8–13: samples collected on October, 8, 2006 (almost 60 days after exposure). Parasitological tests (thick smear or blood culture): samples processed by FIOCRUZ/Bahia and Couto Maia Hospital, Bahia, Brazil); IFAT (Indirect immunofluorescence antibody test): samples processed by LACEN-Bahia, Brazil and FUNED- Minas Gerais, Brazil; ELISA (IgM): samples processed by FUNED- Minas Gerais, Brazil; Elisa with recombinant antigens [20]: samples processed by Edgard Santos University Hospital, Federal University of Bahia, Brazil.

The five family members who did not develop clinical symptoms had repeated negative serological test results for Chagas disease. All five reported to have drunk the same sugar cane juice prepared on August 9, 2006 which was also drunk by the six confirmed cases. However, the five uninfected family members drank the juice more than four hours after it was prepared and two of them had boiled the juice prior to ingestion.

The diagnosis for acute Chagas disease was confirmed using results from positive *T cruzi* parasitological tests: thick smear or blood culture; or a positive serologic test for IgM anti-T-cruzi antibodies: conventional enzyme-linked immunosorbent assay (ELISA), ELISA with recombinant antigens [20], or an indirect immunofluorescence antibody test (IFAT) (Table 1).

The study was approved by the institutional review board of CPqGM-FIOCRUZ, Bahia, Brazil. All patients and/or parents signed a letter of informed consent prior to examination.

### Treatment

All patients were treated after the Chagas diagnosis, which occurred between seven to 14 days and 27 to 37 days after the onset of symptoms in the Macaúbas and Ibipitanga patient groups, respectively. Oral benznidazole 300mg/day for 60 days was prescribed according to the Brazilian Consensus of Chagas Disease [9].

### Eletrocardiogram (EKG) and Two-Dimensional Doppler Echocardiography (ECHO)

The EKG and ECHO were carried out before or shortly after beginning treatment, and 180 days after the end of specific treatment for Chagas disease. The criteria to define EKG alterations were based on the AHA/ACCF/HRS Recommendations for the Standardization and Interpretation of the Electrocardiogram and on the Guidelines of the Brazilian Society of Cardiology on Analysis and Report Issuance Electrocardiographic [21], [22]. The classification of the severity of the valve disease in adults was based on the American College of Cardiology/American Heart Association Practice Guidelines [23] and the quantification of cardiac chamber size and ventricular mass followed the criteria from American Society of Echocardiography's Guidelines and the European Association of Echocardiography [24].

## Results

### Clinical Findings

**Figure 1:**
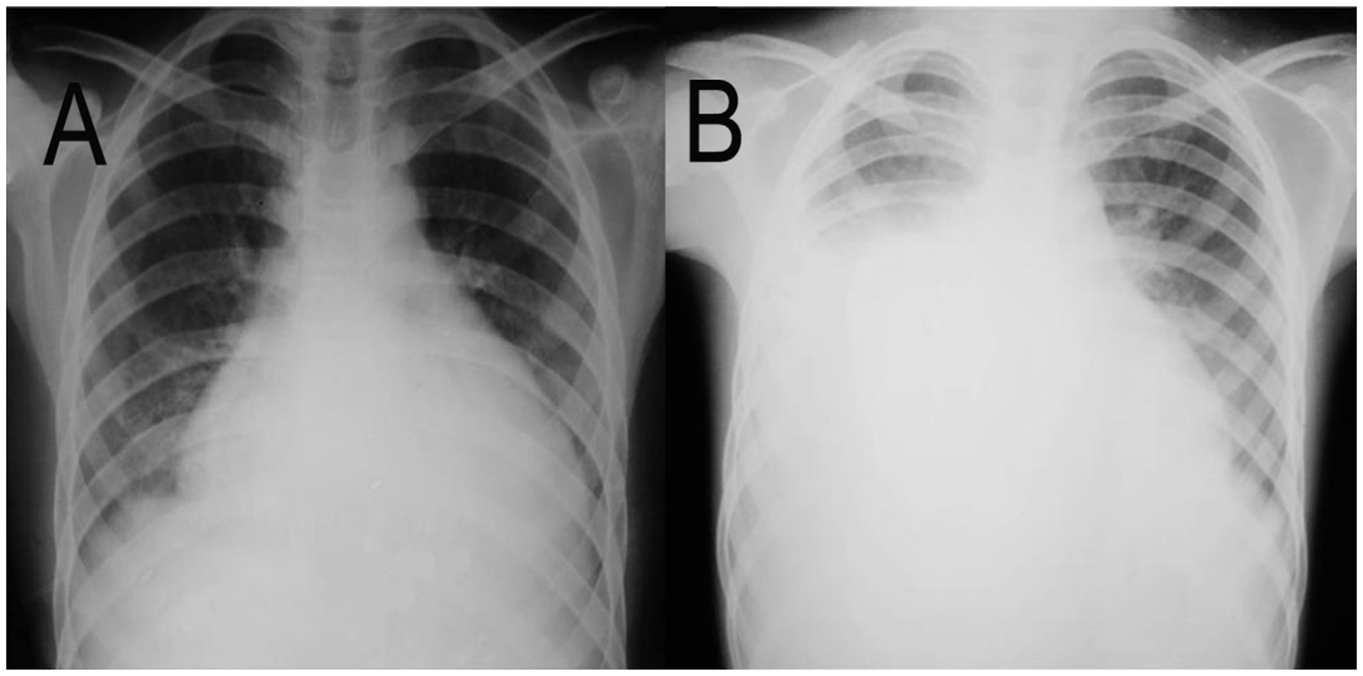
Chest radiographs showing cardiac enlargement and pulmonary congestion in acute Chagas disease. Patient # 6 (A), 16 years old; patient # 7 (B), 09 years old.

The most frequent symptoms in the acute phase were fever (92%) and dyspnea (92%), myalgia (69.2%), periorbital edema (53.9%), headache, systolic murmur (46.2%), nausea, cough, abdominal pain, hepatomegaly (38.5%). Thoracic pain and vomiting were observed in four patients (30.8%), while palpitations were present in three patients (23.1%) (Table 2). Two patients (6, 7) from Macaúbas had heart enlargement, gallop rhythm (3^rd^ sound), tachycardia, hypotension, and cardiac failure, resulting in death. Chest X-rays showed pleural effusion and cardiac enlargement (Figure 1A and B) in both patients. These patients were brothers and were the first in their family to develop the symptoms of this disease. In these cases, the diagnosis of Chagas disease was based on epidemiological findings alone. The mortality rate of Chagas disease, before benznidazole treatment, was 28.6% in Macaúbas and one pregnant woman from Ibipitanga experienced a spontaneous abortion prior to receiving treatment.

**Table 2:** Frequencies of signs and symptoms of thirteen patients with acute Chagas disease from Macaúbas and Ibipitanga, Bahia-Brazil.

Symptoms and signs	N (%)
Fever	12 (92.3)
Dyspnea	12 (92.3)
Myalgia	9 (69.2)
Periorbital edema	6 (46.2)
Headache	6 (46.2)
Cardiac murmur	6 (46.2)
Nausea	5 (38.5)
Cough	5 (38.5)
Abdominal pain	5 (38.5)
Hepatomegaly	5 (38.5)
Thoracic pain	4 (30.8)
Vomits	4 (30.8)
Palpitations	3 (23.1)
Edema in legs	2 (15.4)
Gallop rhythm (third sound)	2 (15.4)
Anasarca	2 (15.4)
Abortion	1 (7.7)
Syncope	1 (7.7)

### EKG and ECHO Alterations

Table 3 displays the most frequent EKG and ECHO findings. EKG data were available for 12 out of 13 patients. At initial presentation, all 12 patients had a disturbance of ventricular repolarization. Right bundle branch block was observed in three out of 12 patients (25%) (cases 2, 3, 6) and sinus bradycardia was observed in cases 2, 12 and 13. Atrial fibrillation was present only in case 4 (8.3%).

**Table 3:** Clinical outcome, electrocardiogram (EKG) and Two-dimensional Doppler Echocardiography (ECHO) of thirteen patients with acute Chagas disease from Macaúbas and Ibipitanga, Bahia, Brazil, after benznidazole treatment.

Patient				Admission		After benznidazole treatment	
ID #	Heart Failure	Age	Gender	EKG 1	ECHO 1	EKG 2¥	ECHO 2¥
1	No	13	F	DVR	Normal	Normal	Normal
2	No	18	M	RBBB, DVR, SB	PE_F_, MR	Normal	Normal
3	No	14	F	RBBB,DVR	PE_F_, MR, SD	DVR	MR
4	No	42	F	DVR, AFib	PE_F_, MR, TR, SD	DVR	MR
5	No	11	M	DVR	Normal	DVR	Normal
6	Yes, death	16	M	RBBB, DVR	Not done	Not done	Not done
7	Yes, death	09	M	Not done	Not done	Not done	Not done
8	No	25	F	DVR	Normal	Normal	Normal
9	Yes	61	M	DVR	PE_F_, EF = 52%	Normal	MR
10	Yes	29	M	DVR	MR, TR, EF = 28%	DVR	Normal
11	No	24	F	DVR	Normal	Normal	Normal
12	Yes	21	M	DVR, SB	MR, EF = 54%	DVR, SB	Abnormal Relaxation
13	No	37	M	DVR, SB	MR, TR	DVR, SB	Normal

Patient #6 and 7 died before evaluation.

**¥:** performed 180 days after the end of benzonidazol treatment.

*MR = Mitral Regurgitation; PE_F_ = Pericardic Effusion; SD = Septum Dyskinesis; DDLV = diastolic Disfunction of Left Ventricule, RBBB = right bundle branch block, DVR = Disturbance of Ventricular Repolarization, AFib = Atrial Fibrillation, SB = Sinus Bradcardia, TI = Tricuspid Regurgitation.

EKG according the AHA/ACCF/HRS 2009 Recommendations for the Standardization and Interpretation of the Electrocardiogram [21] and Guidelines of the Brazilian Society of Cardiology 2009 [22].

ECHO according the ACC/AHA 2006 practice guidelines [23] and ASE committee recommendations [24].

ECHO results were available for 11 out of 13 patients. Seven out of 11 patients had major alterations: a mild degree of mitral regurgitation was observed in six out of 11 patients (54.6%) (cases 2, 3, 4, 10, 12, 13). Pericardial effusion was observed in cases 2, 3, 4, and 9. Tricuspide regurgitation was found in cases 4, 10 and 13 (27.3%), and dyskinetic septum was observed in cases 3 and 4 (18.2%). Ventricular dysfunction with low ejection fraction <55% was present in cases 9, 10 and 12 (27.3%). ECHO findings were normal for cases 1, 5, 8, and 11.

### Effect of Benznidazole Treatment on EKG and ECHO Alterations

No adverse events during treatment with benznidazole were observed. As shown in Table 3, EKG results normalized in five out of 11 patients (91.7%), 180 days after treatment ended. Ventricular repolarization abnormalities persisted in six out of 11 patients (50%) while sinus bradycardia was observed in two patients (16.7%). The atrial fibrillation that was present in case 4 ceased after treatment. Regarding ECHO findings, mitral regurgitation persisted only in cases 3, 4 but disappeared in cases 2, 10, 12, and 13. After treatment, mitral regurgitation was present in case 9. Ventricular function normalized in cases 9, 10 and 12, and pericardial effusion was not present.

## Discussion

Acute Chagas disease caused by oral transmission has been increasingly reported in Brazil and other Latin American countries [5]–[16], [25]. However, few studies describe clinical outcomes after treatment with benznidazole [26]. In this report, the post-treatment clinical evolution of acute Chagas disease in patients from two impoverished rural towns in northeastern Brazil was observed. Oral transmission was determined to be the cause of both micro-outbreaks of acute Chagas disease. In the Macaúbas outbreak, patients were purportedly infected by the ingestion of stored water contaminated by the feces of infested triatomines [8]; while in Ibipitanga, oral transmission was due to the ingestion of sugarcane juice prepared in an abandoned sugarcane mill, where specimens of *T. sordida* contaminated with *T. cruzi* were captured.

Fever and dyspnea were experienced by nearly all patients. Other symptoms and findings indicating myocardial involvement, such as periorbital edema, chest pain and pericardial effusion, were observed in more than one-third of patients in both outbreaks. Hematological and digestive tract symptoms, including gastrointestinal bleeding and gastritis, were not found in our series, but were observed in patients from the Santa Catarina outbreak [6].

Morbidity and mortality rates of severe symptomatic acute Chagas disease are notably higher in children who contract the disease [11]–[12], [27]. Moreover, orally transmitted Chagas disease causes more severe symptoms in acute phases [8]–[11]. Prior to receiving treatment, two children from Macaúbas died as a consequence of heart failure (28.6% mortality rate), while in Ibipitanga, one pregnant woman experienced a spontaneous abortion. In both outbreaks, almost all exposed individuals developed severe manifestations of acute Chagas disease. In the Macaúbas outbreak the attack rate was 100% [8]. In the Ibipitanga outbreak, the six cases had drunk freshly-made sugarcane juice, while the five uninfected family members drank the juice more than four hours after it was prepared and two of them boiled the juice prior to ingestion. These five individuals remained asymptomatic and had repeated negative serological test results for Chagas disease. Insect-derived metacyclic trypomastigotes have specialized mechanisms that allow mucosal invasion. In orally-infected mice, trypomastigotes are able to invade and replicate in the gastric mucosa, causing a systemic infection [28]–[29]. In addition, metacyclic trypomastigotes taken from a patient who was orally infected with acute Chagas disease caused high parasitemia and a high mortality rate in orally-infected mice [30]. The development of myocarditis in acute *Trypanosoma sp.* infection is associated with intense edema of the cardiac fibers and the presence of inflammatory infiltrate containing amastigote forms of *T. cruzi* [4]. High levels of inflammatory cytokines are associated with myocardial damage. A recent study found higher levels of interferon-gamma, tumor necrosis factor-alpha, interleukin-10 and CCL3 in the myocardium of hamsters exhibiting acute symptoms of Chagas disease, when compared to asymptomatic animals [31].

EKG and ECHO abnormalities are frequently observed as a consequence of myocarditis. In the acute phase of Chagas disease, EKG findings may present alterations including low QRS voltage, prolonged PR and/or QT intervals, as well as T-wave changes [4], [17]. Ventricular extra systoles, sinus tachycardia, atrial fibrillation and advanced grade right bundle branch block are all associated with a poor prognosis [27]. In this study, during the acute phase of Chagas disease, ECHO exams were normal in only one-third of patients. Pericardial effusion was observed in 36% of patients. These findings were similar to those observed by Pinto et al during the acute phase of Chagas disease [26].

Six-months after the end of treatment with benznidazole, every patient showed an improvement in clinical symptoms, as well as a decrease in the number of EKG and ECHO abnormalities. Clinical signs of cardiac dysfunction, such as ejection fraction by ECHO, showed improvement in patients with more severe cardiac manifestations (cases 9, 10 and 12). In addition, 45% of patients (5 out of 11) with acute Chagas disease had a normal EKG at the end of treatment. We cannot conclude that improvements in EKG and ECHO findings were directly attributable to therapy, as opposed to the natural course of the disease. Although there is ample information on the clinical evolution of chronic Chagas disease, there are few studies that have evaluated the effect of benznidazole treatment on EKG and ECHO exams in patients with acute Chagas disease who have been followed from initial presentation to convalescence [11], [26]–[27]. In children in the early chronic phase of Chagas disease, after three to four years of follow-up, no conclusive evidence was obtained to indicate that benznidazole treatment, when compared with a placebo, could revert EKG abnormalities [32]–[33]. However, adults with indeterminate and chronic Chagas disease who were treated with benznidazole developed fewer electrocardiographic abnormalities when compared with untreated patients [34]. The present study exclusively involved patients in the acute phase of Chagas disease, for whom treatment is mandatory [8]. As such, it would be unethical to deprive these patients of treatment, making a clinical trial with a placebo control impossible. The efficacy of benznidazole in acute Chagas disease is demonstrated by a reduction in parasite load [35]. The cure rate for parasitological acute Chagas disease ranges from 60 to 80%, depending on patient age, dosage and whether treatment was initiated at the beginning of the infection [36]–[37].

We can conclude that cardiac alterations occur frequently, and are potentially severe, in the acute phase of orally transmitted Chagas disease. Furthermore, EKG and ECHO findings may have an impact on the clinical management of the disease, enabling monitoring of disease progression both during and after benznidazole treatment. Further studies are necessary to evaluate the persistence of EKG and ECHO abnormalities over the long term, as well as morbidity and mortality.
